# Aspect based sentiment analysis datasets for Bangla text

**DOI:** 10.1016/j.dib.2024.111107

**Published:** 2024-11-02

**Authors:** Mahmudul Hasan, Md. Rashedul Ghani, K.M. Azharul Hasan

**Affiliations:** a Department of Computer Science and Engineering, Khulna University of Engineering & Technology, Khulna 9203, Bangladesh; b Department of Computer Science and Engineering, IUBAT— International University of Business Agriculture and Technology, 4 Embankment Drive Road, Sector-10, Uttara Model Town, Dhaka 1230, Bangladesh

**Keywords:** Sentiment analysis, Aspect based sentiment analysis, Bangla sentiment analysis, Opinion mining, Natural language processing

## Abstract

Sentiment analysis is becoming rapidly important for exploring social media Bangla text. The lack of sufficient resources is considered to be an important challenge for Aspect Based Sentiment Analysis (ABSA) of the Bangla language. The ABSA is a technique that divides the text and defines its sentiment based on its aspects. In this paper, we developed a high-quality Bangla ABSA annotated dataset namely BANGLA_ABSA. The datasets are labelled with aspects category and their respective sentiment polarity to do the ABSA task in Bangla. Four open domains namely Restaurant, Movie, Mobile phone, and Car are considered to make the dataset. The datasets are called *Restaurant_ABSA, Movie_ABSA,* Mobile_phone_ABSA, and *Car_ABSA* respectively that contain 801, 800, 975, and 1149 comments. All the comments are either complex or compound sentences. We created the dataset manually and annotated the same by exerting opinions. We organized the dataset as three tuples in Excel format namely 〈*Id, Comment, {Aspect category, Sentiment Polarity}〉*. These data are very important that facilitate the efficient handling of sentiment for any machine learning and deep learning research, especially for Bangla text.

Specifications Table

**Table:** 

Subject	Artificial Intelligence.
Specific subject area	Bangla Natural Language Processing, Sentiment Analysis in Bangla
Type of data	Table (Text/String).
Data collection	The dataset was initially created manually by a large number of people who are undergraduate students. The domains were explained clearly to them and requested to create complex or compound sentences/comments with two aspects and sentiment polarity of the aspect. These types of complex and compound sentences/comments are not commonly found in the web. The initial dataset was divided into three and each part was assigned to three annotators to label the comments into 〈aspect, polarity〉 pair. In case of disagreement of a comment, the majority voting was considered.
Data source location	Institution: Khulna University of Engineering & Technology City/Town/Region: Khulna Country: Bangladesh
Data accessibility	Repository name: Mendeley Data Data identification number: 10.17632/998m4jy3m9.3 Direct URL to data: https://data.mendeley.com/datasets/998m4jy3m9/3 Instructions for accessing these data: By using the URL, anyone can view the datasets publicly.
Related research article	None

## 1 Value of the Data

• Aspect-based Sentiment Analysis (ABSA) is a specific sentiment analysis task that aims to extract the most important aspects of an entity and predict the polarity of each aspect from the text. Bangla is the sixth most spoken language in the world. It is called resource scare language because very few resources are found especially for ABSA. The datasets will enrich the field of Bangla ABSA as well as the Bangla Natural Language Processing (NLP) task.
• One important property of the ABSA dataset is that all the sentences are complex or compound. All the comments of the datasets are labelled into two pairs of aspect category and sentiment polarity which is different from other existing datasets. However, generally only one labelled pair of aspect category and sentiment polarity for a comment or review is found in the other existing sentiment datasets [1,2].
• The datasets can be utilized for developing machine learning and deep learning models to do ABSA tasks for the Bangla text for the applications of sentiment analysis or opinion mining such as business analysis, product review, market research social media monitoring etc.
• In E-commerce, aspects of a product such as price and size are analyzed separately. Therefore, ABSA provides a complete opinion summary at the aspect level, providing detailed sentiment information and offering more precise sentiment insights. Therefore, the proposed dataset can be used for fine-grained sentiment analysis and recommender systems.
• There is a potential for cross-domain use of the dataset in Bangla ABSA tasks. For example, the Mobile_phone_ABSA dataset can be applied particularly for laptop, personal computer, and digital camera related text analysis. These electronic items have similar aspects and sentiment patterns of text such as performance, design, camera, battery, and storage. In the same way, the Car_ABSA dataset can be used for many aspects that are significantly common to cars namely such as motorbikes, motorboats, and other vehicles to develop ABSA tasks for performance, exterior, interior, and accessories. Hence with some domain-specific modifications, the proposed dataset can be used in a variety of domains.

## 2 Background

Natural language processing (NLP) includes sentiment analysis as a key component that becomes essential for interpreting the enormous quantity of content created by users on the internet [3]. It aims to extract sentiments from text, providing insights into people's opinions and attitudes across various topics. With the widespread use of social media, where users spend significant time daily, sentiment analysis has become crucial for understanding public sentiment. Conventional sentiment analysis works focus on overall sentiment at the sentence or document level [4]. This method uses the assumption of a single sentiment towards a single topic, which is often not the case to apply in the actual situation. As a result, recognizing aspect-level opinions and sentiments has gained attention in the past decade [5]. Aspect Based Sentiment Analysis (ABSA) models shift the focus from entire sentences or documents to specific entities or aspects of entities. ABSA task is two types’ namely single and compound where the single ABSA is a part of compound ABSA. While ABSA has seen significant development in languages such as English, European languages, Arabic, and Hindi, but the Bangla language has been relatively underrepresented in sentiment ABSA research. Despite being one of the most spoken languages globally, the scarcity of datasets and resources poses a significant barrier to conducting ABSA research in Bangla [6]. Datasets are created in [1,2] for Bangla ABSA tasks. These data are mostly simple sentence with a single aspect. Although there are few sentences for multiple aspects are present there. Therefore, compound and complex ABSA computation is difficult with the dataset. A dataset for sentiment and emotion classification is developed in [7] which provides a comprehensive insight into consumer behavior and preferences in the context of Bangla text. Once again, most of the sentences are simple sentences with a single aspect.

Recognizing this gap, the objective of this work is to contribute to the enhancement of Bangla language resources by developing datasets specifically tailored for ABSA. By addressing the shortage of resources and datasets, this endeavor seeks to pave the way for more extensive research and development in Bangla sentiment analysis, thereby fostering a deeper understanding of public sentiment and consumer behavior within the Bangla-speaking community.

## 3 Data Description

We have made our datasets openly accessible on a data repository [8]. The dataset is arranged in a folder called “BANGLA_ABSA dataset”, which contains *Car_ABSA, Mobile_phone_ABSA, Movie_ABSA,* and *Restaurant_ABSA* datasets respectively. The *Restaurant_ABSA* contains the comments on restaurant items like food, service, price etc. that shows the sentiment about a restaurant of its customers. The comments are complex or compound sentences with two aspects. For example, the sentence “খাবারটা সুস্বাদু ছিল কিন্তু দাম বেশি ছিল (The food was good but price was high)” has two aspects food (খাবার) and price (দাম). The polarity of the food is positive and the polarity of the price is negative. The dataset is organized as three tuples namely 〈*Id, Comment, {Aspect category, Sentiment Polarity}〉*.

**Fig. 1:**
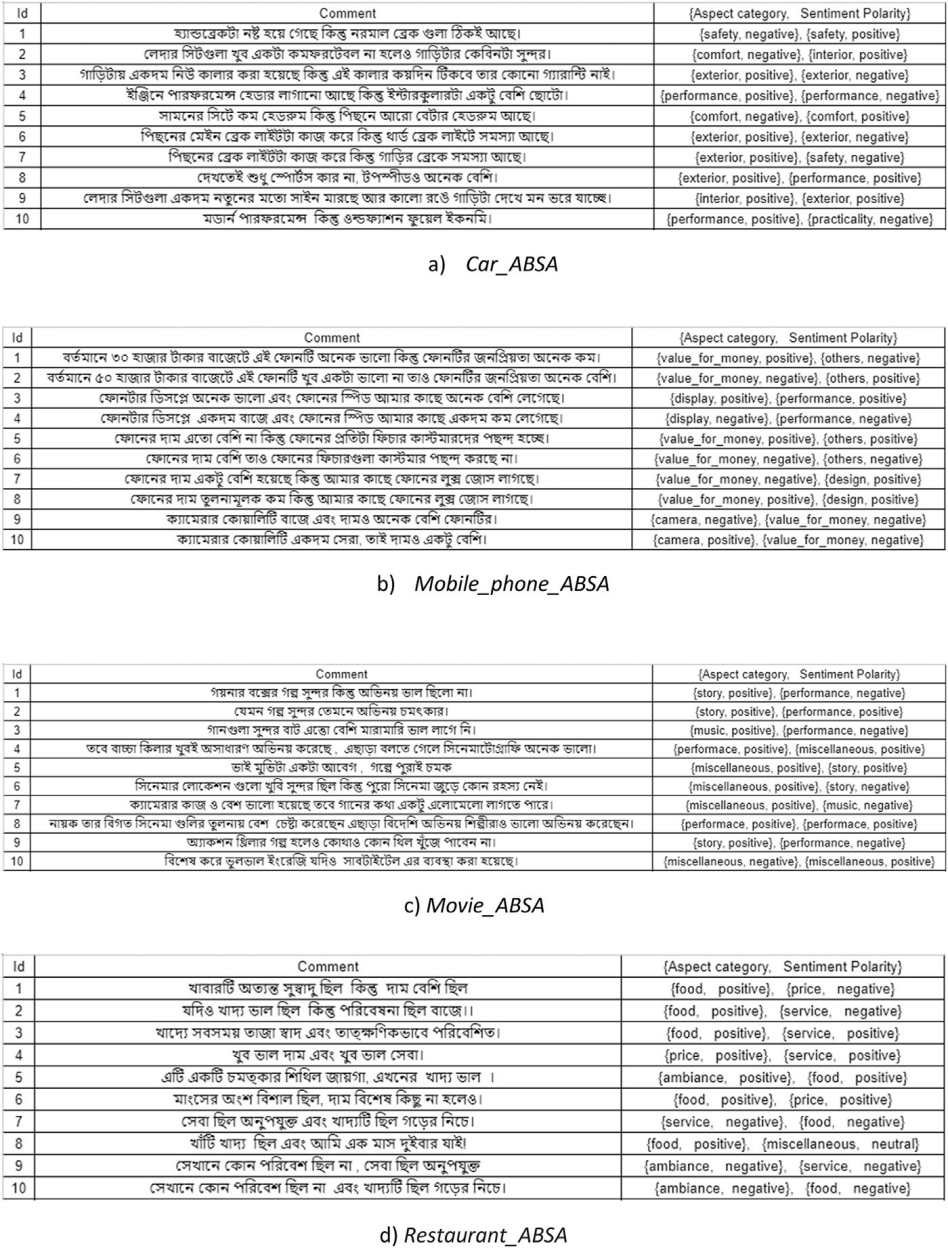
Snapshot of the datasets.

The other three datasets namely *Car_ABSA, Mobile_phone_ABSA,* and *Movie_ABSA* contain user comments about Car, Mobile phone, and Movie respectively. Table 1 presents an overview of the dataset for four different domains. Snapshots of the dataset are shown in Fig. 1. All the comments are either complex or compound sentences with two aspects and two (same or opposite) polarities. The average length (Average number of words) of Car_ABSA, Mobile_phone_ABSA, Movie_ABSA, and Restaurant_ABSA datasets is 10.26, 10.14, 11.78, and 11.22, respectively. The maximum number of words in the dataset of four domains is 19, 20, 35, and 23 respectively. Table 2 shows the statistics of the dataset.

**Table 1:** Overview of the proposed datasets.

Domain name	Aspect category	Polarity	No. of comments
*Car_ABSA*	Performance, Exterior, Interior, Accessories, Comfort, Safety, Practicality, Others	Positive, Negative	1149
*Mobile_ phone_ABSA*	Performance, Design, Display, Camera, Battery, Storage, Value_For_Money, Others	Positive, Negative	975
*Movie_ABSA*	Story, Performance, Music, Miscellaneous	Positive, Negative	800
*Restaurant_ABSA*	Food, Price, Service, Ambiance, Miscellaneous	Positive, Negative	801

**Table 2:** Statistics of proposed datasets.

Dataset	No. of comment	Maximum length (in words)	Minimum length (in words)	Average length (in words)	Maximum length (in characters)	Minimum length (in characters)	Average length (in characters)
*Car_ABSA*	1149	19	5	10.26	128	30	62.13
*Mobile_ phone_ABSA*	975	20	4	10.14	109	23	59.42
*Movie_ABSA*	800	35	4	11.78	195	23	69.63
*Restaurant_ ABSA*	801	23	4	11.22	144	23	65.43

## 4 Experimental Design, Materials and Methods

The dataset has one important property that each of the text/sentence is a complex or compound sentence. Each of the text has two aspects with two sentiment polarities. It is unusual to get such text in the web or bog. Initially the dataset was created manually by a large number of people who are mostly undergraduate (BSc. engineering) students. The domains of the dataset are described clearly and requested to create complex or compound sentences within the domain with two aspects and two sentiment polarities (positive or negative). For example the text “the food was good but price was high” has two aspects food and price and two sentiment polarities namely 〈food, positive〉 and 〈price, negative〉. All the comments did not meet the ABSA criteria such as complex or compound sentences. These data were pre-processed manually to meet the criteria of ABSA. The initial dataset was divided into three and each part was assigned to three annotators to annotate the text into 〈aspect, polarity〉 pair manually. These annotators are also undergraduate students. The majority voting was considered to adopt a 〈aspect, polarity〉 text annotating in the dataset.

Table 3 shows an example of annotation. After a majority vote, the comment was finally annotated with a food aspect and positive polarity, and a price aspect and negative polarity. After annotation of the data, the dataset has the scope of improvement. After annotation of the comments the data is still noisy. The following preprocessing was applied to the proposed datasets after annotation.

• Some of the punctuation characters such as (‘:’, ’;’) are removed as these punctuations are not very meaningful for the ABSA task. On the other hand some punctuations such as ‘, ’, ’!’, ’?’, ’|’ are used to make meaningful sentences.

• Stop words are inconsequential words with little value in processing specific tasks. Stop words that are used frequently in a text but are not important for the ABSA task. For example, ছিল, অতএব, এই, করবে, তবু, হোক etc. are the stop word for Bangla ABSA task and are removed.
• For example, the punctuation of the text “এই গাড়িটা অপূর্ণ একটা প্যাকেজ: আরামদায়ক হলেও গতি একদমই নাই।” can be removed to generate “এই গাড়িটা অপূর্ণ একটা প্যাকেজ আরামদায়ক হলেও গতি একদমই নাই।” The stop words can also be removed to process the text “গাড়িটা অপূর্ণ প্যাকেজ আরামদায়ক হলেও গতি একদমই নাই।”. We used the stop words listed in [9].

**Table 3:** An example of annotation.

Comment: যদিও খাবার ভাল ছিল কিন্তু পরিবেষনা ছিল বাজে। (Although the food was good but service was bad)	
Annotator	{Aspect category, Sentiment Polarity}
A1	{food, positive}, {service, negative}
A2	{food, positive}, {service, negative}
A3	{food, positive}, {service, negative}

To evaluate the proposed datasets, some supervised machine-learning models namely Naive Bayes (NB) , Logistic Regression (LR) , and Support Vector Machine (SVM) were applied using the proposed datasets separately and achieved satisfactory performance for ABSA. Among the three machine learning models, SVM gained the highest F1 score. The experimental results for the four different datasets are presented for aspect category classification and sentiment polarity classification in Table 4, Table 5 respectively. For aspect category classification, the F1 scores were 83.13 %, 93.63 %, 95.60 %, and 97.00 % respectively. For sentiment polarity classification, the scores were 84.00 %, 82.50 %, 83.67 %, and 95.67 % respectively. From the experimental results with important machine learning models, it is evident that they are producing good and stable results both for aspect detection and sentiment polarity tasks. Hence, we can conclude that the dataset is well balanced for ABSA tasks for Bangla text.

**Table 4:** Aspect category classification performance on the proposed datasets.

Dataset	Model	Precision (%)	Recall (%)	F1-score (%)
*Car_ABSA*	NB	82.13	73.38	76.00
	LR	81.50	78.50	79.63
	SVM	**83.75**	**82.38**	**83.13**
*Mobile_Phone_ABSA*	NB	90.63	77.00	81.13
	LR	93.63	86.38	89.13
	SVM	**95.63**	**92.63**	**93.6**3
*Movie_ABSA*	NB	55.20	55.60	55.20
	LR	98.40	87.00	91.80
	SVM	**99.00**	**93.00**	**95.60**
*Restaurant_ABSA*	NB	99.00	80.17	86.00
	LR	98.67	85.00	90.67
	SVM	**99.33**	**95.00**	**97.00**

**Table 5:** Sentiment polarity classification performance on the proposed datasets.

Dataset	Model	Precision (%)	Recall (%)	F1-score (%)
*Car_ABSA*	NB	78.50	78.50	78.50
	LR	81.50	80.50	81.00
	SVM	**84.50**	**84.00**	**84.00**
*Mobile_Phone_ABSA*	NB	79.50	78.00	79.00
	LR	80.00	79.00	79.50
	SVM	**82.50**	**82.00**	**82.50**
*Movie_ABSA*	NB	58.33	60.00	59.33
	LR	**98.33**	74.33	81.33
	SVM	94.33	**78.33**	**83.67**
*Restaurant_ABSA*	NB	92.33	76.00	81.00
	LR	95.67	86.67	90.00
	SVM	**99.33**	**92.33**	**95.67**

Although the dataset is developed for specific domains but the dataset, in a broad sense, represents consumer behavior and urban experiences, ignoring any rural or other socioeconomic viewpoints and can be applied to other domains with some little modification that can assist the sentiment expressions in a wider context. A comparative assessment of the proposed dataset with [[1], [2]] is presented in Table 6 applying SVM model. The result shows superior precision, recall and F1-score for the proposed dataset.

**Table 6:** Comparison of the dataset for SVM model.

Dataset	Precision	Recall	F1-score
Cricket [1]	0.71	0.22	0.34
Restaurant [1]	0.77	0.30	0.38
BAN-ABSA [2]	0.74	0.66	0.69
Car_ABSA	0.84	0.82	0.83
Mobile_Phone_ABSA	0.96	0.93	0.94
Movie_ABSA	0.99	0.93	0.96
Restaurant_ABSA	0.99	0.95	0.97

## Limitations

The limitations of the datasets are as follows:

• The dataset is domain dependent namely Car, Mobile phone, Movie, and Restaurant.
• The length of the comments (in words) of the proposed datasets is not long. The maximum length is 35 words. Hence the long dependency may not be captured.

## Ethics Statement

The authors do not have any ethical issues for developing the dataset. The datasets are fully anonymous and the data redistribution policies. The identity of the commenter has been obscured to protect their privacy.

## CRediT authorship contribution statement

**Mahmudul Hasan:** Conceptualization, Methodology, Data curation, Validation, Writing – original draft. **Md. Rashedul Ghani:** Data curation, Validation, Writing – original draft. **K.M. Azharul Hasan:** Supervision, Writing – review & editing.

## Data Availability

Mendeley DataAspect Based Sentiment Analysis Datasets for Bangla Text (Original data).
